# Potential contribution of PEP carboxykinase-dependent malate dismutation to the hypoxia response in *C. elegans*

**DOI:** 10.1038/s41467-023-39510-5

**Published:** 2023-07-04

**Authors:** Rosina Comas-Ghierra, Laura Romanelli-Cedrez, Gustavo Salinas

**Affiliations:** 1 Worm Biology Lab, Institut Pasteur de Montevideo, Montevideo, Uruguay; 2 Department of Biosciences, Facultad de Química, Universidad de la República, Montevideo, Uruguay

**Keywords:** Respiration, Gene regulation, Cell signalling

**arising from** M. Vora et al. *Nature Communications* 10.1038/s41467-022-33849-x (2022)

Oxygen is vital for animals, driving energy production through oxidative phosphorylation. Humans, for instance, cannot survive more than a few minutes without respiring. Yet, animals face hypoxia, either environmental (low oxygen availability), functional (e.g., during extreme exertion) or as a result of pathological processes such as cancer or infection. Thus, metabolic adaptations to changes in oxygen levels are key for animal survival. These adaptations require oxygen sensors that trigger a complex rewiring of gene expression and cellular metabolism. In animals, changes in gene expression in response to hypoxia are driven by the transcription factor HIF (hypoxia-inducible factor, HIF-1 in *C. elegans*)^1^. HIF-1 stability and activity are controlled by the oxygen sensor HIF-prolyl hydroxylase EGLN (EGL-9 in *C. elegans*). In aerobiosis, EGL-9 hydroxylates HIF-1, using oxygen as a substrate, targeting HIF-1 for VHL-1-mediated proteasomal degradation^2^. In hypoxia, no hydroxylation occurs, and VHL-1 does not recognize HIF-1, which remains intact and activates the hypoxia-induced response. In humans, the reprogramming of metabolism driven by HIF-1 leads to a metabolic shift towards glycolysis and lactate production, as oxygen-dependent oxidative phosphorylation is reduced^1^.

In a recent article, Vora et al. capitalized on this mechanism of action and analyzed *C. elegans* mutants lacking *egl-9* (and therefore HIF-1 is constitutively active) or *hif-1* under aerobic conditions. They used RNA-seq and ChIP-seq to analyze the gene expression in these mutants^3^. The authors found that HIF-1 upregulates the expression of hundreds of genes. The striking upregulation of the cytosolic PEP carboxykinase, *pck-1*, and cytosolic malate dehydrogenase, *mdh-1*, led the authors to suggest that hypoxia promotes gluconeogenesis. This metabolic pathway converts pyruvate to glucose through an opposite but not identical chain of reactions to that of glycolysis. A key gluconeogenesis detour is catalyzed by cytosolic PEP carboxykinase, which converts oxaloacetate into PEP, coupled to GTP hydrolysis. Cytosolic malate dehydrogenase, on the other hand, generates oxaloacetate from malate.

**Fig. 1:**
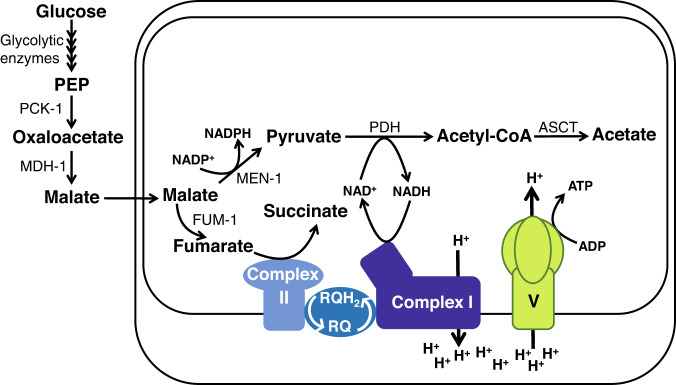
Energy metabolism under hypoxia. Several animal lineages such as worms, bivalves, and annelids harvest energy from malate dismutation under hypoxia. In this pathway malate is generated from glucose through glycolytic enzymes up to phosphoenolpyruvate (PEP), which is converted to oxaloacetate by PEP carboxykinase (PCK-1) and then to malate by malate dehydrogenase (MDH-1). Malate enters the mitochondria and undergoes dismutation, resulting in the production of succinate and acetate. The oxidative branch converts malate to pyruvate catalyzed by the malic enzyme (MEN-1), and pyruvate to acetyl-CoA by pyruvate dehydrogenase (PDH-1). Acetyl-CoA is then converted to acetate by acetyl-succinyl-CoA transferase (ASCT). The reductive branch converts malate to fumarate, catalyzed by fumarase (FUM-1), and fumarate is subsequently reduced to succinate by complex II. These two reactions function in reverse to the conventional TCA. Thus, in the absence of oxygen, fumarate is the final electron acceptor. The reversal of complex II is facilitated by the lipidic electron transporter rhodoquinone (RQ, rhodoquinone, oxidized form; RQH_2_, rhodoquinol, reduced form) whose redox potential is intermediate between the redox potential of fumarate/succinate and NAD^+^/NADH, allowing electrons to flow from NADH to fumarate in an alternative electron transport chain that pumps protons, keeps the redox balance and drives ATP synthesis by ATP synthase (complex V). Acetate and succinate are excreted as end products of malate dismutation; in some organisms, succinate can be further decarboxylated to propionate. The rounded rectangles correspond to the inner and outer mitochondrial membranes.

In addition to the interpretation that hypoxia promotes gluconeogenesis, we put forward a complementary explanation for the observed increase in *pck-1* and *mdh-1* expression. Worms, including *C. elegans*, annelids, and bivalves subject to hypoxic conditions harvest energy through an alternative abbreviated electron transport chain (ETC) in which fumarate, not oxygen, is the final electron acceptor and rhodoquinone, instead of ubiquinone, is the lipidic electron transporter^4,5^ (Fig. 1). In this alternative ETC, complex I oxidizes NADH and reduces rhodoquinone while pumping protons to drive ATP synthesis by the mitochondrial ATP synthase (complex V)^6^. In contrast to the conventional complex II, which functions as succinate dehydrogenase, in this ETC the standard redox potential of the rhodoquinone/rhodoquinol pair facilitates complex II activity as fumarate reductase, leading to rhodoquinol oxidation. This ETC is sustained by malate dismutation, a mitochondrial pathway adapted to anaerobic functioning^7^.

Malate dismutation involves a reductive branch from malate to succinate, and an oxidative branch that converts malate to acetate (Fig. 1). In the reductive branch, malate is converted to fumarate by the tricarboxylic acid cycle (TCA cycle) enzyme fumarase, and then to succinate, by the alternative complex II, both working in reverse to the conventional TCA cycle. Succinate could be excreted or; in some worms, further decarboxylated to propionate, which is then excreted^7,8^. In the oxidative branch, malate is decarboxylated to pyruvate by the malic enzyme, generating NADPH, and then pyruvate is decarboxylated yielding Acetyl-CoA, which is finally converted to acetate by acetyl-succinyl CoA transferase (ASCT) and excreted. The NADH generated in the oxidative reactions can be oxidized to NAD^+^ by complex I. The key metabolite of this pathway is malate, which derived from incomplete glycolysis that generates PEP and then uses PCK-1 and MDH-1. In this pathway, MDH-1 and PCK-1 function in the opposite direction to gluconeogenesis^7,9^.

In several parasitic worms, e.g., *Fasciola hepatica*, *Hymenolepis* spp., and *Ascaris* spp., malate dismutation is the main glucose catabolic pathway in hypoxia, although lactic acid fermentation has also been described^10,11^. In these organisms, the PEP carboxykinase/pyruvate kinase ratio is highest in the adult worm, the lifecycle stage subject to the host gut environmental hypoxia. This increase is accompanied by the excretion of organic acids derived from malate dismutation. More recently, the transcriptomic analysis of *Hymenolepis microstoma* transition from the cysticercoid to the adult stage revealed a significant increase in transcriptomic abundances of genes coding for key enzymes of malate dismutation, cytosolic PEP carboxykinase, and cytosolic malate dehydrogenase, revealing a major metabolic shift in the normoxia/hypoxia transition^12^. The metabolomic analysis presented by Vora et al. indicated an increase in metabolites shared by both gluconeogenesis and malate dismutation pathways, such as malate and fumarate. The exometabolome would provide further insights to discern which of the pathways were activated by HIF-1. Vora et al. also infer that the glyoxylate cycle would be used to synthesize oxaloacetate to feed the gluconeogenesis pathway. The glyoxylate cycle requires the β-oxidation of fully reduced fatty acids, which in turn demands oxygen. Yet, it is possible that low levels of oxygen may be sufficient to temporarily promote a baseline of fatty acid oxidation for the glyoxylate cycle.

Mutants lacking *hif-1* or *pck-1* showed reduced hypoxia-starvation survival, which could be partially restored by PEP, but not by pyruvate. This would argue for a role of PEP carboxykinase in gluconeogenesis, but PEP is also a direct source of ATP since can be converted to pyruvate, releasing an ATP. In any case, this does not exclude the importance of PCK-1 in malate dismutation. New experiments using oxaloacetate and different labeled metabolites would be informative to dissect the complexity of the intermediary metabolism in wild-type and mutant strains.

It is important to note that the RNA-seq and ChIP-seq were performed in aerobic conditions, which is a different context from hypoxia, and corresponds to the whole organism. Tissue-specific pathway compartmentalization has not been extensively explored in *C. elegans*. It is possible that some tissues may require gluconeogenesis for generating reducing power through the pentose phosphate pathway while other tissues may need malate dismutation for harvesting energy from glucose, particularly under hypoxic conditions. Rhodoquinone, whose biosynthesis was recently elucidated^13,14^, is a key evolutionary innovation to harvest energy in animals that face hypoxia, doubling the energy obtained by lactic acid fermentation. The alternative ETC that functions with rhodoquinone consumes fumarate, which as far as it is known from well-studied organisms, derives from malate dismutation, and requires PEP carboxykinase and malate dehydrogenase to sustain malate synthesis. These enzymes could have been co-opted for malate dismutation and serve additional roles to the conventional one in gluconeogenesis. Nevertheless, the interplay between PEP carboxykinases (neural/muscle PCK-1 and epidermal/intestinal/muscle PCK-2) and ETC could be complex in *C. elegans*. A recent article has shown that genetic mutations in complex II subunit A upregulate the protein accumulation of PCK-2 and PCK-1^15^, suggesting potential metabolic redirection away from the conventional mitochondrial ETC.

Finally, it is important to note that stabilization of HIF-1 under aerobic conditions by *egl-9* or *vhl-1* mutants, although having overlapping effects with hypoxia, may not fully mimic the conditions of hypoxia. This is something to be aware of while interpreting the original manuscript by Vora et al. and this viewpoint. To sum up, we wanted to highlight other relevant metabolic adaptations to hypoxia absent in mammals.
